# Supplementary material for: A descriptive analysis of 2020 California Occupational Safety and Health Administration covid-19-related complaints
Source: SSM Popul Health. 2021 Dec 29;17:101016. doi: 10.1016/j.ssmph.2021.101016 (PMC8714613; doi:10.1016/j.ssmph.2021.101016)
Supplement: Multimedia component 1 [file mmc1.docx]

**Supplemental Material 1**. Industry and Complaint Variable Coding

**Industry**: variable coding using NAICS industry name

1=Health Care and Social Assistance

2=Retail Trade

3=Manufacturing

4=Accommodation and Food Services

5=Transportation and Warehousing

6=Agriculture, Forestry, Fishing and Hunting

7=Other (Public Administration; Construction; Wholesale Trade; Other Services (except Public Administration); Management of Companies and Enterprises; Professional, Scientific, and Technical Services; Educational Services; Arts, Entertainment, and Recreation; Finance and Insurance; Real Estate and Rental and Leasing; Information; Utilities; Mining, Quarrying, and Oil and Gas Extraction)

**Business Type**: variable coded using Cal/OSHA “ownership_type”

1 = local government

2 = private sector

3 = state government

**Complaint Type**: variable coded using Cal/OSHA “severity_subject”

1 (Health) = Discrimination O-Health, I-Health, O-Health, Discrimination S-Health

2 (Safety) = O-Safety, I-Safety, S-Safety

3 = (Health & Safety) = O-Health;O-Safety, O-Health;S-Health, O-Safety;S-Health, S-Health;S-Safety

**Complaint Severity**: variable coded using Cal/OSHA “severity_subject”

1 (Low) = Discrimination O-Health, I-Health, I-Safety, O-Health, O-Safety, O-Health;O-Safety

2 (High) = O-Health;S-Safety, O-Safety;S-Health, Discrimination S-Health; S-Health;S-Safety

**Formality**: variable coded using Cal/OSHA “formality”

1 = formal (reported by a named employee)

2 = nonformal (reported by an anonymous employee)

**Inspection**: variable coded using Cal/OSHA “do_insp”

0 (No) = do_insp = N; NA

2 (Yes) = do_insp = Y

**Reason for Inspection**: variable coded using Cal/OSHA “reason_for_insp” and restricted to “do_insp=Y”

1 (Required) = Valid Formal Complaint Submitted; Formal Complaint Alleges Recordkeeping Deficiencies; Alleged Imminent Danger

2 (Follow-up) = Inadequate/No ER Response to Injury - Includes Employee Dispute; Inspection of Employer is Scheduled/in Progress; Abatement Follow-up/Monitoring Inspection

3 (AD Discretion) = AD Discretion

**Penalty**: variable coded using Cal/OSHA “proposed_penalty” and restricted to “do_insp=Y”

0 (No) = proposed_penalty=0

1 (Yes) = proposed_penalty>0
