# Supplementary material for: A descriptive analysis of 2020 California Occupational Safety and Health Administration covid-19-related complaints
Source: SSM Popul Health. 2021 Dec 29;17:101016. doi: 10.1016/j.ssmph.2021.101016 (PMC8714613; doi:10.1016/j.ssmph.2021.101016)
Supplement: Multimedia component 2 [file mmc2.docx]

| **Supplemental Material 2**. Distribution of Cal/OSHA COVID-19-related inspections characteristics by industry, 2020. | | | | | | | | | |
| --- | --- | --- | --- | --- | --- | --- | --- | --- | --- |
|  | Total | Health Care & Social Assistance | Retail Trade | Manufacturing | Accommodation & Food Services | Transportation & Warehousing | Agriculture, Forestry, Fishing & Hunting | Other | P-value |
|  | *row n (%)* | | | | | | | | |
| Inspections |  |  |  |  |  |  |  |  | 0.000 |
| Total | 627 (100.0) | 153 (24.4) | 98 (15.6) | 81 (13.0) | 63 (10.0) | 41 (6.5) | 32 (5.1) | 159 (25.4) |  |
|  |  |  |  |  | *column n (%)* |  |  |  |  |
| Business Type |  |  |  |  |  |  |  |  | 0.000 |
| Local | 22 (3.5) | 4 (2.6) | 0 (0.0) | 0 (0.0) | 0 (0.0) | 3 (7.3) | 0 (0.0) | 15 (9.4) |  |
| Private | 587 (93.6) | 146 (95.4) | 98 (100.0) | 81 (100.0) | 63 (100.0) | 37 (90.3) | 32 (100.0) | 130 (81.8) |  |
| State | 18 (2.9) | 3 (2.0) | 0 (0.0) | 0 (0.0) | 0 (0.0) | 1 (2.4) | 0 (0.0) | 14 (8.8) |  |
| Complaint Type |  |  |  |  |  |  |  |  | 0.062 |
| Health (H) | 560 (89.3) | 141 (92.2) | 89 (90.8) | 67 (82.7) | 61 (96.8) | 37 (90.2) | 30 (93.8) | 135 (84.9) |  |
| Safety (S) | 28 (4.5) | 2 (1.3) | 4 (4.1) | 5 (6.2) | 1 (1.6) | 2 (4.9) | 0 (0.0) | 14 (8.8) |  |
| H & S | 39 (6.2) | 10 (6.5) | 5 (5.1) | 9 (11.1) | 1 (1.6) | 2 (4.9) | 2 (6.2) | 10 (6.3) |  |
| Reason |  |  |  |  |  |  |  |  | 0.000 |
| Required | 266 (42.4) | 66 (43.1) | 36 (36.7) | 29 (35.8) | 17 (27.0) | 23 (56.1) | 13 (40.6) | 82 (51.6) |  |
| Follow-up | 101 (16.1) | 17 (11.1) | 24 (24.5) | 8 (9.9) | 20 (31.7) | 6 (14.6) | 0 (0.0) | 26 (16.3) |  |
| AD's Discretion | 260 (41.5) | 70 (45.8) | 38 (38.8) | 44 (54.3) | 26 (41.3) | 12 (29.3) | 19 (59.4) | 51 (32.1) |  |
| Formality |  |  |  |  |  |  |  |  | 0.376 |
| Formal | 245 (39.1) | 59 (38.6) | 32 (32.7) | 32 (39.5) | 20 (31.7) | 20 (48.8) | 12 (37.5) | 70(44.0) |  |
| Nonformal | 382 (60.9) | 94 (61.4) | 66 (67.3) | 49 (60.5) | 43 (68.3) | 21 (51.2) | 20 (62.5) | 89 (56.0) |  |
| Severity |  |  |  |  |  |  |  |  | 0.000 |
| Low | 500 (79.7) | 91 (59.5) | 88 (89.8) | 72 (88.9) | 56 (88.9) | 37 (90.2) | 27 (84.4) | 129 (81.1) |  |
| High | 127 (20.3) | 62 (40.5) | 10 (10.2) | 9 (11.1) | 7 (11.1) | 4 (9.8) | 5 (15.6) | 30 (18.9) |  |
| Penalty |  |  |  |  |  |  |  |  | 0.005 |
| No | 595 (94.9) | 138 (90.2) | 95 (96.9) | 75 (92.6) | 63 (100.0) | 38 (92.7) | 29 (90.6) | 157 (98.7) |  |
| Yes | 32 (5.1) | 15 (9.8) | 3 (3.1) | 6 (7.4) | 0 (0.0) | 3 (7.3) | 3 (9.4) | 2 (1.3) |  |
|  |  |  |  |  |  |  |  |  |  |
| *Note: Abbreviation: AD = Area Director; Chi-square test compares complaint characteristic across the six industry categories.* | | | | | | | | | |
